# A new way of analyzing tooth movement using universal coordinate system geometry single point superposition in a 3D model

**DOI:** 10.1590/2177-6709.28.4.e232333.oar

**Published:** 2023-10-09

**Authors:** Rodrigo Xavier Silveira de SOUZA, Gustavo Almeida Silveira de SOUZA, João Pacheco COLARES, Tânia Mara de Souza IANNI, Cláudia Silami de MAGALHÃES, José Alejandro GUERRERO-VARGAS, Carina Cristina MONTALVANY-ANTONUCCI, Soraia MACARI

**Affiliations:** 1 Federal University of Minas Gerais, Faculty of Dentistry, Department of Restorative Dentistry (Belo Horizonte/MG, Brazil); 2 Federal University of São João del Rei, Department of Telecommunications and Mechatronics Engineering (Ouro Branco/MG, Brazil); 3 Universidad ECCI, Faculty of Engineering, Department of Mechanical Engineering (Bogotá, Colombia)

**Keywords:** Digital models, 3-dimensional diagnosis and treatment planning, Reproducibility of results

## Abstract

**Introduction:**

Superposing 3D models is an imminent need. However, current methods rely on marking multiple points on the maxilla and mandible, which could increase point marking and overlapping errors.

**Objective:**

This study aimed at developing a method for superimposing 3D models of the maxillary and mandibular arches with Autodesk Inventor® engineering software, using a single universal coordinate system (UCS) point superposition.

**Methods:**

A total of 104 STL (stereolithography) models of the maxillary and mandibular arches exported from My iTero® platform were retrospectively selected, in which T0 and T1 were the initial and refinement periods, respectively (n=26 per group). The X, Y, and Z coordinates associated with a single point in each arch were inserted into the models with SlicerCMF® software for model orientation. The arch models with UCS registration were transferred to Autodesk Inventor® for superimposition and to measure tooth movements performed during Invisalign® treatment. Arch expansion, intrusion and rotation were analyzed by two examiners. The statistics were performed using intraclass correlation coefficients (ICC), Dahlberg’s formula, and t-test (p<0.05).

**Results:**

A reliable method of superimposing 3D digital models using a single UCS point in the maxilla and mandible was developed. ICC showed excellent intra- and inter-examiner correlation (ICC>0.90). A systematic error was not found concerning linear and angular measurements (<1mm and <1.5°, respectively). Digital dental movements could be analyzed, including arch expansion, dental intrusion, and tooth rotation.

**Conclusions:**

The developed method was proven reliable and reproducible for superimposing 3D models of the maxillary and mandibular arches by using UCS system.

## RESUMO

**Introdução:**

A sobreposição de modelos 3D é uma necessidade iminente. No entanto, os métodos atuais dependem da marcação de múltiplos pontos na maxila e na mandíbula, o que pode aumentar a incorporação de erros no processo de sobreposição.

**Objetivo:**

O objetivo desse estudo foi desenvolver um método para sobrepor modelos 3D das arcadas superior e inferior utilizando o *software* de engenharia Autodesk Inventor®, por meio da marcação de um único ponto em cada arcada, usando o sistema de coordenadas universal (UCS).

**Métodos:**

No total, 104 modelos STL das arcadas superior e inferior exportados da plataforma My iTero® foram selecionados retrospectivamente, onde T0 foi o período inicial e T1, o de refinamento (n=26 por grupo). As coordenadas X, Y e Z associadas a um único ponto em cada arcada foram inseridas nos modelos usando o *software* SlicerCMF®. Os modelos com os pontos UCS demarcados foram transferidos para o *software* Autodesk Inventor® para realizar a sobreposição e medir os movimentos dentários realizados durante o tratamento com Invisalign®. Os movimentos de expansão, intrusão e rotação foram analisados por dois examinadores. A análise estatística foi realizada usando os coeficientes de correlação intra-classe (ICC), fórmula de Dahlberg e teste *t* (p<0,05).

**Resultados:**

Foi desenvolvido um método confiável de sobreposição de modelos digitais 3D usando um único ponto UCS na maxila e mandíbula. O ICC apresentou excelente correlação intra e inter-avaliadores (ICC>0,90). Não foi encontrado erro sistemático nas medidas lineares e angulares (<1mm e <1,5°, respectivamente). Os movimentos dentários puderam ser analisados por meio do método proposto, incluindo expansão da arcada, intrusão e rotação dentária.

**Conclusão:**

O método desenvolvido provou ser confiável e reprodutível para sobreposição de modelos 3D das arcadas superior e inferior usando o sistema UCS com marcação de ponto único.

## INTRODUCTION

Digital model superimposition is mainly used for observation of occlusal changes caused by craniofacial growth,^1^ ^,^ ^2^ analysis of accuracy and predictability of orthodontic treatment with aligners,^3^ ^,^ ^4^ and evaluation of orthodontic tooth movement.^5^ ^-^ ^8^ The use of three-dimensional (3D) digital models for orthodontic outcome analysis has advantages over other methods, such as less exposure to radiation, quality of image visualization and lower cost.^7^

Several methods seek reliability for overlapping 3D models by demarcating points in the maxilla and mandible that are stable and do not move during orthodontic treatment.^6^ ^,^ ^9^ ^-^ ^11^ Studies that looked for superimposition points in the maxilla observed that the most stable is situated in the posterior part of the incisor papilla and the palatal rugae.^10^ ^,^ ^12^ ^,^ ^13^ Regarding the mandible, solid points that can be used for superimposition in 3D models are scarce.^7^ ^,^ ^11^ Thus, there is a need for studies to establish a stable point for better accuracy in mandibular superimposition.^14^

Dental software programs have been developed to assist orthodontists in treatment planning, but all have shown limitations and difficulties when used in research.^15^ ^-^ ^17^ Autodesk Inventor^®^is an engineering software program that uses the Computer-Aided Design/Computer-Aided Manufacturing (CAD/CAM) system to build products and items, and to help understand how a product or body behaves under certain conditions. It uses the Universal Coordinate System (UCS) that defines the orientation of the Cartesian axes X, Y, and Z in three-dimensional space, associated with a single point mark on the part/model with high accuracy. Autodesk Inventor^®^was used to evaluate implant accuracy.^18^ Due to its advantages, the use of Autodesk Inventor^®^in the superimposition of 3D models in Dentistry might bring reliability and agility to the studies of three-dimensional movements in the maxillary and mandibular arches.^18^

Understanding the step-by-step of superposition is mandatory for analyzing 3D models.^19^ However, it is known that this is not simple and that the researcher or clinician can face many difficulties. In this sense, to overcome these obstacles and make 3D analyses simpler, more effective and cheaper, this study aimed to provide a step-by-step protocol of 3D models superimposition of the maxillary and mandibular arches, by marking a single point using the UCS geometry with SlicerCMF^®^and Autodesk Inventor^®^software.

## MATERIAL AND METHODS

**Figure 1:**
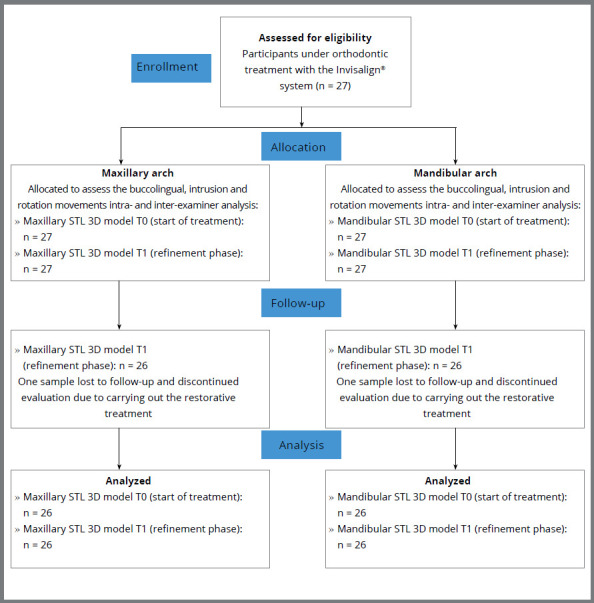
CONSORT flow diagram.

This retrospective observational study was approved by the Ethics and Research Committee of the Federal University of Minas Gerais (CAAE: 48546321.4.0000.5149) and the Brazilian Clinical Trials Registry (ReBEC, # RBR-7df547h). Sample size calculation (Supp. Table 1) and inclusion and exclusion criteria were determined ( Suppl. Table 2). The sample was composed of 104 STL (Stereo-lithographic or Standard Triangle Language) models from 27 participants, treated with the Invisalign^®^system (Align Technology, Santa Clara, CA, USA) and exported from my iTero^®^platform (https://clincheck.invisalign.com Align Technology, Santa Clara, CA, USA) ( Fig 1). One participant carried out a restorative treatment and lost the follow-up in the refinement phase, thus a total of 26 participants were included in the study (Fig 1).

**Table 1:** Results of the intra-examiner agreement in the buccolingual translation, intrusion and rotation movements.

**Tooth**	**n**	Measure 1 (RS)		Measure 2 (RS)		Type of movement				
		Mean	SD	Mean	SD	ICC	95% CI	D	P	BA
						Buccolingual translation (mm)				
Maxillary right canine	26	0.250	0.225	0.254	0.233	0.993	0.962-0.992	0.038	0.952	-0.003
Maxillary right first premolar	26	0.412	0.363	0.394	0.329	0.885	0.743-0.948	0.155	0.857	0.017
Maxillary right second premolar	26	0.446	0.413	0.468	0.396	0.916	0.813-0.962	0.158	0.849	-0.039
Maxillary right first molar	26	0.329	0.227	0.341	0.249	0.887	0.747-0.949	0.105	0.853	-0.012
Maxillary right second molar	26	0.365	0.257	0.371	0.267	0.955	0.900-0.980	0.075	0.937	-0.005
Maxillary left canine	26	0.259	0.187	0.268	0.213	0.882	0.736-0.947	0.091	0.863	-0.009
Maxillary left first premolar	26	0.434	0.460	0.535	0.608	0.853	0.671-0.934	0.277	0.503	-0.100
Maxillary left second premolar	26	0.539	0.557	0.483	0.418	0.878	0.728-0.945	0.228	0.682	0.056
Maxillary left first molar	26	0.356	0.271	0.379	0.251	0.840	0.643-0.928	0.135	0.756	-0.022
Maxillary left second molar	26	0.237	0.160	0.294	0.211	0.823	0.599-0.922	0.108	0.283	-0.057
Mandibular right canine	26	0.270	0.200	0.328	0.291	0.816	0.583-0.919	0.151	0.410	-0.062
Mandibular right first premolar	26	0.323	0.308	0.290	0.269	0.990	0.978-0.996	0.041	0.678	0.033
Mandibular right second premolar	26	0.448	0.532	0.424	0.525	0.990	0.978-0.996	0.064	0.869	0.024
Mandibular right first molar	26	0.372	0.353	0.359	0.379	0.977	0.950-0.990	0.305	0.900	-0.001
Mandibular right second molar	26	0.269	0.199	0.267	0.199	0.979	0.951-0.991	0.032	0.966	0.002
Mandibular left canine	26	0.317	0.303	0.305	0.306	0.971	0.936-0.987	0.262	0.888	-0.003
Mandibular left first premolar	26	0.298	0.330	0.250	0.269	0.983	0.962-0.992	0.281	0.562	0.038
Mandibular left second premolar	26	0.341	0.435	0.292	0.319	0.969	0.931-0.986	0.331	0.642	0.048
Mandibular left first molar	26	0.377	0.345	0.304	0.279	0.978	0.950-0.990	0.289	0.404	0.060
Mandibular left second molar	26	0.236	0.197	0.178	0.150	0.948	0.885-0.977	0.064	0.239	0.050
						Rotation (degrees)				
Mandibular right central incisor	26	6.920	5.485	6.347	4.999	0.961	0.911-0.983	1.418	0.697	0.319
Mandibular right lateral incisor	26	6.414	6.234	6.258	7.248	0.979	0.953-0.991	1.342	0.934	-0.093
Mandibular right canine	26	8.654	8.487	8.307	8.529	0.987	0.969-0.994	1.376	0.883	-0.318
Mandibular left central incisor	26	7.043	5.512	6.322	5.211	0.969	0.932-0.986	1.316	0.626	0.478
Mandibular left lateral incisor	26	5.466	4.847	5.258	5.020	0.960	0.908-0.982	1.343	0.880	-0.002
Mandibular left canine	26	7.623	6.156	7.054	6.121	0.970	0.932-0.987	1.134	0.741	0.287
						Intrusion (mm)				
Mandibular right central incisor	26	0.231	0.343	0.211	0.311	0.996	0.992-0.998	0.028	0.818	0.012
Mandibular right lateral incisor	26	0.243	0.326	0.229	0.280	0.991	0.980-0.996	0.039	0.869	0.005
Mandibular right canine	26	0.155	0.250	0.147	0.224	0.995	0.989-0.998	0.023	0.905	0.007
Mandibular left central incisor	26	0.242	0.344	0.223	0.310	0.995	0.989-0.998	0.033	0.830	0.010
Mandibular left lateral incisor	26	0.279	0.313	0.253	0.284	0.989	0.976-0.995	0.044	0.753	0.016
Mandibular left canine	26	0.207	0.340	0.197	0.304	0.995	0.989-0.998	0.031	0.908	0.002

SD = Standard deviation. ICC = Intraclass Correlation Coefficient. CI = Confidence Interval. D = Dahlberg. BA = Bland-Altman. Mm = millimeters. *p* < 0.05.

**Table 2:** Results of the inter-examiner agreement in the buccolingual translation, intrusion and rotation movements.

**Tooth**	**n**	Examiner 1 (RS)		Examiner 2 (JP)		Type of movement				
		Mean	SD	Mean	SD	ICC	95% CI	D	P	BA
						Buccolingual translation (mm)				
Maxillary right canine	26	0.257	0.224	0.288	0.263	0.980	0.957-0.991	0.047	0.648	-0.031
Maxillary right first premolar	26	0.412	0.363	0.394	0.329	0.887	0.743-0.948	0.155	0.855	0.017
Maxillary right second premolar	26	0.412	0.363	0.394	0.329	0.916	0.813-0.962	0.319	0.857	-0.039
Maxillary right first molar	26	0.329	0.227	0.341	0.249	0.887	0.747-0.949	0.105	0.853	-0.012
Maxillary right second molar	26	0.365	0.257	0.371	0.267	0.955	0.900-0.990	0.074	0.937	-0.005
Maxillary left canine	26	0.259	0.187	0.268	0.213	0.882	0.736-0.947	0.091	0.863	-0.009
Maxillary left first premolar	26	0.434	0.460	0.535	0.608	0.853	0.671-0.934	0.277	0.503	-0.100
Maxillary left second premolar	26	0.539	0.557	0.483	0.418	0.878	0.729-0.945	0.228	0.682	0.056
Maxillary left first molar	26	0.356	0.271	0.379	0.251	0.840	0.643-0.928	0.135	0.756	-0.022
Maxillary left second molar	26	0.237	0.160	0.294	0.211	0.823	0.599-0.922	0.108	0.283	-0.057
Mandibular right canine	26	0.270	0.200	0.328	0.291	0.816	0.583-0.919	0.151	0.410	-0.062
Mandibular right first premolar	26	0.328	0.356	0.333	0.344	0.809	0.573-0.914	0.194	0.956	-0.005
Mandibular right second premolar	26	0.498	0.566	0.474	0.588	0.859	0.679-0.938	0.281	0.884	0.0332
Mandibular right first molar	26	0.375	0.347	0.387	0.381	0.857	0.675-0.937	0.183	0.900	-0.011
Mandibular right second molar	26	0.286	0.200	0.244	0.297	0.910	0.791-0.961	0.091	0.867	-0.006
Mandibular left canine	26	0.360	0.352	0.381	0.338	0.883	0.735-0.949	0.156	0.830	-0.016
Mandibular left first premolar	26	0.404	0.443	0.402	0.444	0.975	0.942-0.989	0.112	0.987	0.001
Mandibular left second premolar	26	0.376	0.440	0.400	0.527	0.988	0.973-0.995	0.103	0.861	-0.055
Mandibular left first molar	26	0.382	0.354	0.427	0.377	0.978	0.952-0.990	0.081	0.656	-0.045
Mandibular left second molar	26	0.240	0.209	0.293	0.249	0.943	0.871-0.975	0.078	0.412	-0.044
						Rotation (degrees)				
Mandibular right central incisor	26	6.706	5.343	6.557	5.005	0.956	0.896-0.981	1.476	0.922	0.149
Mandibular right lateral incisor	26	6.859	7.583	7.102	7.348	0.986	0.966-0.994	1.275	0.913	-0.565
Mandibular right canine	26	8.244	6.978	8.356	7.165	0.980	0.952-0.992	1.361	0.958	-0.112
Mandibular left central incisor	26	6.467	4.645	6.816	5.827	0.966	0.918-0.986	1.467	0.824	-0.968
Mandibular left lateral incisor	26	5.715	4.847	6.291	5.270	0.981	0.953-0.992	1.136	0.704	-0.862
Mandibular left canine	26	8.046	6.402	8.480	6.969	0.982	0.957-0.993	1.342	0.829	-0.818
						Intrusion (mm)				
Mandibular right central incisor	26	0.193	0.316	0.188	0.289	0.992	0.983-0.987	0.036	0.956	0.004
Mandibular right lateral incisor	26	0.255	0.320	0.233	0.383	0.937	0.859-0.972	0.120	0.826	0.021
Mandibular right canine	26	0.115	0.343	0.131	0.350	0.987	0.970-0.994	0.056	0.867	-0.016
Mandibular left central incisor	26	0.238	0.325	0.236	0.349	0.990	0.979-0.0996	0.045	0.987	0.001
Mandibular left lateral incisor	26	0.281	0.311	0.261	0.295	0.989	0.976-0.995	0.045	0.809	0.020
Mandibular left canine	26	0.214	0.337	0.227	0.389	0.927	0.837-0.967	0.132	0.897	-0.013

SD = Standard deviation. ICC = Intraclass Correlation Coefficient. CI = Confidence Interval. D = Dahlberg. BA = Bland-Altman. Mm = millimeters. *p* < 0.05.

**Figure 2:**
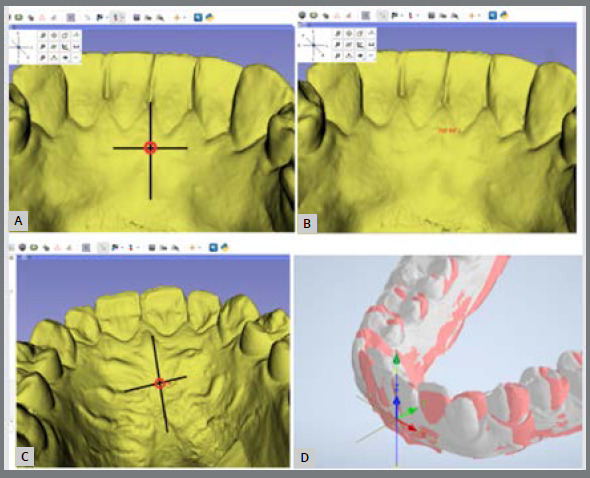
Mandibular and maxillary landmark position using vertical and horizontal lines to define the point of interest position. A) Mandibular landmark position; B) geometric coordinates generated after marking the point; C) maxillary landmark position; D) mandibular superposed models.

**Figure 3:**
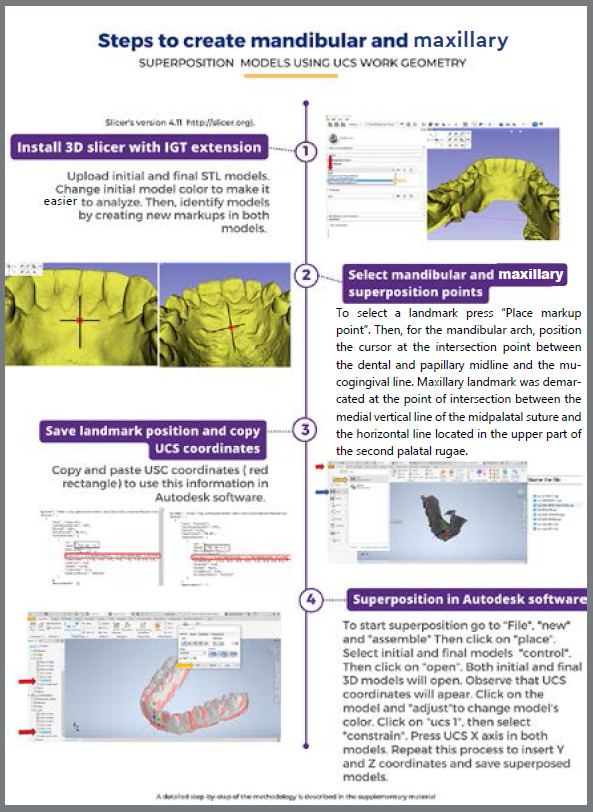
Flowchart indicating a summary of the step-by-step procedure for marking points on the maxilla and mandible using Slicer 3D^®^ software and superimposing models in Autodesk Inventor^®^ software.

**Figure 4:**
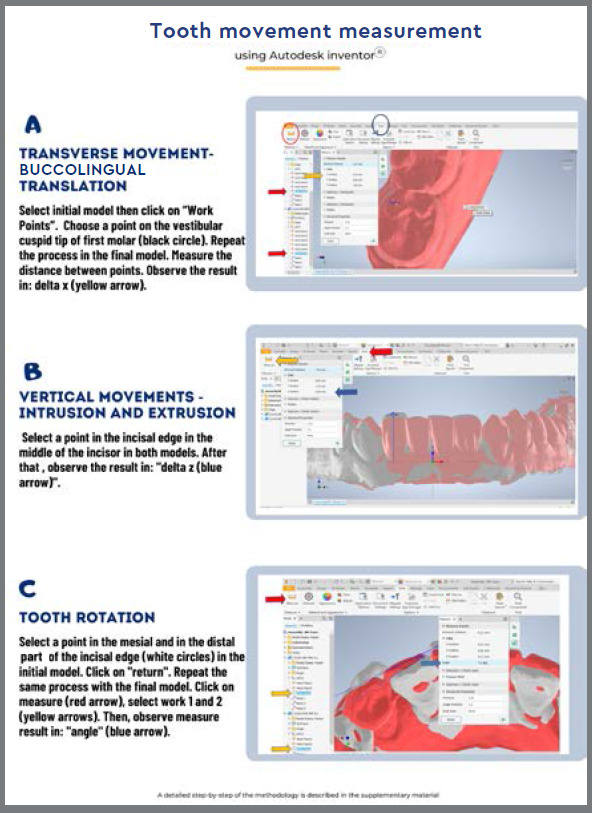
The image represents tooth movements measurement: A) Buccolingual translation; B) Vertical movements (intrusion and extrusion); C) Rotation movements.

The maxillary and mandibular arches were scanned using an iTero^®^scanner (model Element, S/N: RTC2018 W06A228) by two operators, under the same conditions, at two time-points: T0 (start of treatment) and T1 (refinement phase). Models superimposition and analysis were performed using SlicerCMF^®^(version 4.11; http://slicer.org) and Autodesk Inventor^®^software. The first step was to determine the maxillary or mandibular landmarks in the 3D SlicerCMF^®^software, to generate the UCS coordinate system numbers in T0 and T1 models. In the maxilla, the reference point was created with a vertical line passing through the medial region of the palate suture and a horizontal line passing through the upper region of the second palate rugae. The intersection between these lines was considered the region of interest and a landmark was created (Fig 2). To standardize the superposition process in the mandible, a vertical line passing through the midline region was developed with a horizontal line passing through the mucogingival junction (Fig 2). The intersection between these lines was considered the region of interest and a landmark was developed. The X, Y, and Z coordinates were then set from these maxillary and mandibular landmarks, establishing stable references in space in T0 and T1 models. Afterward, the X, Y, and Z coordinates were copied and used in Autodesk Inventor^®^(Fig 3) to promote an overlap between the geometric points in T0 and T1 models. After superimposing models, tooth movement was measured in Autodesk Inventor^®^(Fig 4 ). The 3D movements of buccolingual translation of maxillary and mandibular canines, premolars and molars were analyzed, and the intrusion and rotation movements of the mandibular incisors and canines were measured. The intra- and inter-examiner calibration was performed. Examiner 1 (RS) repeated the measurements twice with a 15-day interval (intra-examiner) and Examiner 2 (JP) performed these measurements once (inter-examiner). A detailed description of the methodology was prepared and can be seen in the supplementary material (Suppl. Table 3).

### STATISTICAL ANALYSIS

The results were expressed as mean ± standard deviation. Intra- and inter-examiner agreements were calculated using an intraclass correlation coefficient (2-way random, single measurement, absolute agreement) and Bland-Altman plot,^1^ using the SPSS (IBM SPSS Statistics version 19.0, Inc., New Armonk, NJ, EUA) and GraphPad Prism softwares (version 8.0 for Mac, La Jolla, California, USA), respectively. For the assessment of systematic error, the Dahlberg formula was used. For systematic error evaluation, linear measurements should not exceed 1 mm and angular measurements should not exceed 1.5°.^20^ The steps for model analysis were performed by two examiners (RS and JP, inter-examiner). The first examiner performed the steps twice, with a 15-day interval between examinations (intra-examiner). ICC values of 0.9 to 1 were considered excellent, 0.6 to 0.7 were good, 0.4 to 0.5 were considered reasonable, and less than 0.4 were categorized as poor.^20^ Student’s t-test was used to analyze the differences between the groups, and *p* < 0.05 was considered statistically significant. Statistical analysis was performed using the program GraphPad Prism (version 8.0 for Mac, La Jolla, California, USA). 

## RESULTS

### SUPERPOSITION OF THE 3D MODEL WAS REACHED BY STANDARDIZING LANDMARK LOCATION IN THE MAXILLA AND MANDIBLE AND USING UCS GEOMETRY

**Figure 5:**
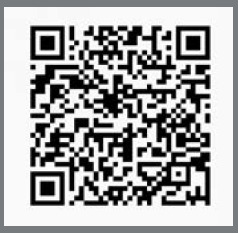
“Step-by-step” video.

The study generated a step-by-step guide that can be accessed in the supplementary material (Suppl. Table 3) and a video to help researchers perform the superimposition of STL models. The first step is the download of SlicerCMF^®^and Autodesk Inventor^®^softwares. Both softwares are freely available for students. Afterward, the step-by-step and the video should be followed to help the analysis. The superposition process in the maxilla and mandible was standardized using UCS geometry, single point in 3D STL models. The 3D buccolingual translation and intrusion movements were measured in millimeters and rotation movement in degrees. The buccolingual translation movement was measured as the distance between the buccal cusps of canines, and mesiobuccal cusps of premolars and molars on the X-axis in millimeters (mm). The vertical movement of the mandibular canines and incisors was measured from the tip of the canine cusps to the middle of the incisal edges. Displacements in the gingiva direction (intrusion movement) were considered with a positive sign (+); Displacements in the incisal occlusal direction (extrusion movement) were considered with a negative sign (-). The amount of vertical movement was observed in the Z-axis in millimeters (Suppl. Table 3 and Fig. 5). 

The “Step-by-step” video can be watched through the QR code above (Fig 5).

### INTER- AND INTRA-EXAMINER MEASURES SHOWED A HIGH DEGREE OF AGREEMENT

Intra- and inter-examiner measures showed a high degree of agreement, analyzed by ICC and Bland-Altman statistical tests, indicating that the methodology of 3D models superposition was effective (Tables 1 and 2).

Measurements demonstrated excellent intra-examiner agreements within the linear measures, with mean errors smaller than 0.07 mm (the largest mean intra-examiner difference was found for the buccolingual movement of the left mandibular first molar: 0.377 ± 0.345 mm [first measure] and 0.304 ± 0.279 mm [second measure]) (Table 1). For the rotation movement, the mean difference was smaller than 0.72° in the left mandibular central incisor (7.043 ± 5.512° [first measure] and 6.322 ± 5.211° [second measure]) (Table 1). Inter-examiner agreements were adequate within the linear measures, with a difference smaller than 0.06 mm (the largest intra-examiner measure was found for the buccolingual movement of the left maxillary second premolar: 0.539 ± 0.557 mm [first measure] and 0.483 ± 0.418 mm [second measure]) (Table 2). For the rotation movement, the mean error was smaller than -0.58° in the left mandibular lateral incisor (7.15 ± 4.847° [first measure] and 6.291 ± 5.270° [second measure]) (Table 2). 

In the linear and rotation measures, Dahlberg values were always smaller than 1 mm and smaller than 1.5°, respectively, demonstrating that no systematic error was found for intra- and inter-examiners (Tables 1 and 2).

## DISCUSSION

The present study was the first to use the association between the SlicerCMF^®^software and the Autodesk Inventor^®^engineering program, using the basic principle of the Universal Coordinate System (UCS). This technique allowed us to determine a single point in the region of the palatine rugae, in the maxilla, and a single point in the lingual region of the mandible, at the mucogingival junction. The results proved to be reliable and reproducible for superimposing 3D orthodontic models of the maxilla and mandible in STL format. In addition, this software is free of charge and easily accessible by the clinical and scientific community, which reduces the financial investment for carrying out research. 

Since Broadbent^21^ and Baumrind, Frantz^22^ investigations on head-film measurements, there has been a challenge to determine an accurate method to set up landmarks to improve treatment planning, description, and predictability. Point identification error is a systematic problem and over the years several studies have proposed different methods to control it.^8^ ^,^ ^12^ ^,^ ^19^ ^,^ ^22^ ^-^ ^24^ The search for stable structures that are not influenced by orthodontic movement has been the main prerogative of current studies.^7^ Even in cases of orthodontic aligners, in which no movement of posterior teeth is planned, making them supposedly immobile, tooth movement is observed.^3^ In this study, we standardized a method to establish landmark points on the maxilla and mandible, to reduce this identification error. In the present method, the points were created by the intersection of two lines (vertical and horizontal), both traced on anatomical structures. Furthermore, this landmark generated by UCS geometry promotes better three-dimensional control of the point location and a more reliable superimposition. 

Studies have pointed out that both the palatal rugae region and the mucogingival region are stable for model superimposition, using a wide area of the maxilla and mandible, respectively.^1^ ^,^ ^7^ ^-^ ^12^ ^,^ ^19^ ^,^ ^24^ ^-^ ^26^ The greatest difficulties in superimposing STL models have been in the mandible.^7^ ^,^ ^13^ ^,^ ^19^ One of the points used that proved stable for mandibular superimposition is the Torus, located on the lingual alveolar surface; however, this is a structure that is not present in all patients^11^ - thus, its use was discarded in the present research. The region of the mucogingival junction has been chosen for 3D superimpositions in the mandible because it is easy to locate and because it is stable and does not change,^19^ even during intrusion and extrusion movements of the teeth or with mandibular growth.^1^ ^,^ ^19^ ^,^ ^24^ ^,^ ^25^ Usually in the literature, these points are demarcated along the mucogingival junction from the first molar on the right side to the first molar on the left side, totaling 13 marks on average.^1^ ^,^ ^19^ ^,^ ^24^ The enhanced number of marks might increase the chance of errors and possibly decrease the reliability of the method. The solution to this impasse in the present study was to use the Cartesian axes associated with a single point at the mucogingival junction in the mandibular region. Through the demarcation of a single point in each model, the three Cartesian axes (X, Y, and Z) were generated and the software aligned these axes in T0 and T1 models, creating infinite points of spatial superposition with stability and reproducibility, as it is a stable and accessible structure present in all cases.^1^ ^,^ ^7^ ^,^ ^19^ ^,^ ^24^

In the maxilla, studies have shown that the rugae regions, especially the second and third rugae, are stable regions for superimposition.^1^ ^,^ ^11^ ^,^ ^24^ ^,^ ^25^ In the current study, the Cartesian axes with a single point in the medial region of the second rugae was used for superimposition of the maxillary models. This area is considered safe because it is not affected by tooth movement,^8^ ^,^ ^10^ ^,^ ^19^ thus decreasing the chance of errors during similar points marking.

Several studies seek to increase the reliability of superposition by searching for spatial reference planes using manual tools for the elaboration of the coordinates.^6^ ^,^ ^11^ ^,^ ^12^ ^,^ ^16^ ^,^ ^19^ ^,^ ^27^ The use of only one point in each 3D model was possible due to the use of the UCS coordinate system. Through UCS in Autodesk Inventor^®^, it was possible to automatically match the Cartesian axes by reading the coordinates of the points in the two models, T0 and T1, and thereby evaluating the displacements of the X (buccolingual displacement), Y (mesiodistal displacement) and Z (vertical displacement) coordinates. Thus, it was possible to observe each variation individually, without approximation mechanisms or manual interference. This method is an essential tool for many precision operations in engineering and can be used in Dentistry, reducing the amount of work required for the superimposition of 3D models.

Autodesk Inventor^®^is commonly used in bioengineering for stress and strain assessment, which requires accurate positioning of the models before mechanical analyses.^28^ ^,^ ^29^ In the present research, this software was fundamental to ensure the correct positioning of the models, which is essential in studies for 3D models superimposition. Autodesk Inventor^®^, as well as other CAD/CAM software, is compatible with several 2D and 3D file extensions, especially the so-called “neutral formats”, such as Standard Triangle Language (STL), and Parasolid (x_t).^24^ The use of this software proved to be very useful in this study, since the maxilla and mandible scans performed in Invisalign^®^orthodontic treatments use the STL format. Thus, it was possible to easily manipulate the models using Autodesk Inventor^®^2D and 3D design tools. 

The software used in our research was the freely available SlicerCMF^®^, which is commonly used in Dentistry and has demonstrated its accuracy and reliability.^19^ It has been used for superimposing 3D models of the maxilla, despite using several points on the palatal rugae,^19^ ^,^ ^30^ and for superimposing points on the mucogingival region of the mandible, using 13 points as a reference for superimposition.^1^ ^,^ ^19^ Herein, we used the SlicerCMF^®^for single point demarcation on both T0 and T1 models. The superimposition was not performed by this software as it requires more than one point for this purpose.^19^ In this study, the points in the lingual mucosa gingival region were easily visualized and demarcated, and registered through the association with the X, Y, and Z coordinates generated by the software itself.

In the present research, the treatment was focused on mild to moderate malocclusions, with duration of eight months between T0 and T1. After the treatment with the aligners, there were few changes in the adjacent gingival tissues; however, none of them could hinder the identification of similar points or structures in T0 and T1, allowing the proper superimposition of the 3D models. The same anatomical structures used in this research can be visualized in more severe malocclusions, showing that there are no limitations to the use of the new superimposition method proposed in this study.^13^ ^,^ ^24^

Considering the ICC of each movement, high values in the linear movements expressed in millimeters were obtained. The lowest value for ICC was for the rotation, expressed in degrees. The reason was the high sensitivity of Autodesk Inventor^®^for angular measurements, especially for values smaller than 5°. During insertion of the points on the incisal edges, minimal displacements in the buccolingual direction within the incisal edge could alter the reading of the angle formed by the two lines (T0 and T1), especially when the STL mesh was enlarged in the models. A different situation was found in the linear measurements, as small horizontal displacements did not affect the result so significantly, due to the size of the measurements. This was an exploratory study, and future studies may consider linear measurement error with a threshold of less than 1 mm and angular measurements of less than 1.5°. Regarding the systematic error, a study stated that less than 1 mm and 1.5° for linear and angular,^20^ respectively, are acceptable, but in the present study, the magnitude of these values may mean clinically relevant movements. 

The STL models were obtained retrospectively by scanning exclusively with iTero^®^, as carried out in other studies,^31-33^ and the spatial position of the models was established by this program automatically. Our focus was to standardize the methodology by choosing cases treated with the same align system, as well as using STL files obtained from the same scanner brand and handled by two operators. We believe that these precautions make the standardization of the methodology more effective. In future studies, another scanner brand may be used to evaluate if this is a factor that influences the spatial positioning of the models. 

Regarding the limitation of the current study, we need to point out that superposing models and measuring tooth movement was not possible in cases in which restorative or periodontal procedures were performed during the orthodontic treatment. A total of 27 participants were selected for the study. One patient was lost to follow-up and discontinued evaluation due to carrying out restorative treatment in T1; so the study was carried out with the remaining 26 participants who had agreed to participate. Another important point is the non-comparison between our method and others in the literature, to assess the accuracy of our systematization. Our main objective was to describe this accessible method that has proven to be effective for superimposing models, and it will be necessary to analyze the accuracy and predictability of this new method as compared with others.^1^ ^,^ ^19^ Thus, further studies are needed to determine the level of accuracy of the methodology.

Inexperience with engineering software can become a real challenge and limitation for dental researchers. However, in this study a very detailed step-by-step procedure was developed explaining complex and unfamiliar commands, which may become easy and intuitive after professional training. After the high agreement and reproducibility results achieved with this program, it may become a new and useful diagnostic tool for outcome assessment and orthodontic treatment predictability within all clinicians’ reach.

## CONCLUSIONS

We can conclude that an effective method for superimposing 3D models using UCS geometry was created, using the 3D SlicerCMF^®^and Autodesk Inventor^®^free softwares. This model was effective in promoting the superposition of the maxillary and mandibular models and in measuring tooth movements, such as tooth expansion, rotation, intrusion, and extrusion.

## Supplemental Table 1. Sample size calculation. The sample size calculation for comparative studies that involve the comparison of two groups (inter- or intra examiner) was performed to verify the number of patients to be used in the research. To determine the value of N an equation comparing two means was used, as previously described by Eng (2003)1. Thus, the formula for calculating the sample size for a reliable estimate of the population mean is given bellow.

[Table t1app]

**Table t1app:**

Equation: $N\;=\;4\delta^{2}{\left(Zcrit\;+Zpwr\right)}^{2}/D^{2}$ Where: N = total number of individuals in the sample δ = standard deviation of the studied variable. Zcrit = normal standard deviation of the selected significance criterion. Zpwr: normal standard deviation of the selected statistical power. D = Minimum expected difference between the 2 means.
Rerefence for equation development: As a reference for the expected value of standard deviation of the mean and the minimum expected difference between 2 means, values referring to the three-dimensional (3D) dental changes (in millimeters) measured in the anteroposterior (A-P) plane of the right first premolar and right canine from the article by Garib et al., (2021)^2^ were used (page 16 of the article, Table), as they were one of the most unstable biological variables found in this study. The standard deviation found for the A-P in the right canine was 1.39; while the difference between the means of A-P measurements from the right canine and the right first premolar was 0.4489 [(-0,41) - 0.26]. A significance criterion of 0.05 (normal standard deviation = 1.96) and statistical power of 0.80 (normal standard deviation = 0.842) were selected based on the article Eng (2003)^1^
Solving the equation with these values provides: $N\;=\;4\;x\;{\left(1.39\right)}^{2}x\;{\left(1.96\;+\;0.842\right)}^{2}/\;{\left(\left(-0,41\right)\;–\;0.26\right]}^{2}$ N=47.83
Participants: As indicated in the equation for sample calculation, the value of N corresponds to the sum of the 2 groups being compared. Thus, the predicted value of N for each experimental group is approximately 23.9 (N = 47.83 divide for 2 groups). Thus, the predicted N value was rounded to 24 patients and an additional of 10% (26.4 patients per group, rounded to 27 participants) was added planning possible losses during the orthodontic treatment. A total of 27 participants were selected for the study. One sample lost to follow-up and discontinued evaluation due to carrying out the restorative treatment in T1 and the study remained with 26 participants that agreed to participate.

References

1. Eng J. Sample size estimation: how many individuals should be studied? Radiology. 2003;227(2):309-13.doi:10.1148/radiol.2272012051

2. Garib D, Miranda F, Massaro C, Lauris JRP, Yatabe MS, Janson G, et al. Three-dimensional mandibular dental changes with aging. American journal of orthodontics and dentofacial orthopedics : official publication of the American Association of Orthodontists, its constituent societies, and the American Board of Orthodontics. 2021;159(2):184-92.doi:10.1016/j.ajodo.2019.12.021

## Supplemental Table 2. Inclusion and exclusion criteria and inter- e intra- examiner’s calibration

[Table t2app]

**Table t2app:**

**Inclusion criteria:** patients who had Class I, II and III malocclusion with moderate inferior crowding of up to 4 mm, change in overbite (from 3 to -5 mm), permanent dentition, presence of all teeth up to the second molars, aged 18 to 45 years.
**Exclusion criteria:** patients who had restorations in the teeth moved during treatment, non-cooperating patients with craniofacial syndromes or anomalies, and those with signs or symptoms of inflammation in the periodontal tissues. Three cases were discarded, two because the mesh was distorted for unknown reasons and one because a composite resin restoration was inserted in a tooth after movement altering the dental proportion, resulting 11 pairs of STL models. All participants were treated by the same professional in a private clinic in Belo Horizonte, Minas Gerais, Brazil and the cases were selected retrospectively.
**Inter e intra examiner’s calibration:** To evaluate the calibration of the main examiner (RS) with the software, a total of 22 measurements were taken, after superimposition 10 on posterior teeth in the bucco-lingual direction of canines, first and second premolars, first and second molars mandibular and maxillary represented by the X axis, in millimeters; 6 measurements in the vertical direction on anterior teeth of canines and mandibular incisors, movement represented by the Z axis in millimeters; and, 6 measurements on anterior teeth for the mesio-distal rotation, carried out by mandibular and maxillary incisors and canines, analyzed in degrees. The superimposition of 11 STL models were performed, totaling 242 measurements in 286 teeth, in both phases T0 (initial) and T1 (refinement), and repeated 2 times with a 15-day interval between them. To evaluate the accuracy and reliability, the same measurements were repeated by two examiners (RS- and GS) of the 11 cases. The two examiners did not participate in the orthodontic treatment.

## Supplemental Table 3: Step by step process to choose mandibular and maxillary superimposition points using UCS working geometry in initial and final STL models.

[Fig f3app]

[Fig f4app]

[Fig f5app]

[Fig f6app]

[Fig f7app]

[Fig f8app]

[Fig f9app]

[Fig f10app]

[Fig f11app]

[Fig f12app]

[Fig f13app]

[Fig f14app]

[Fig f15app]

[Fig f16app]

[Fig f17app]

[Fig f18app]

[Fig f19app]

[Fig f20app]

[Fig f21app]

[Fig f22app]

[Fig f23app]
